# Acute and Postacute COVID-19 Outcomes Among Immunologically Naive Adults During Delta vs Omicron Waves

**DOI:** 10.1001/jamanetworkopen.2023.1181

**Published:** 2023-02-28

**Authors:** Margaret K. Doll, Alpana Waghmare, Antje Heit, Brianna Levenson Shakoor, Louise E. Kimball, Nina Ozbek, Rachel L. Blazevic, Larry Mose, Jim Boonyaratanakornkit, Terry L. Stevens-Ayers, Kevin Cornell, Benjamin D. Sheppard, Emma Hampson, Faria Sharmin, Benjamin Goodwin, Jennifer M. Dan, Tom Archie, Terry O’Connor, David Heckerman, Frank Schmitz, Michael Boeckh, Shane Crotty

**Affiliations:** 1 Department of Population Health Sciences, Albany College of Pharmacy & Health Sciences, Albany, New York; 2 Division of Infectious Diseases, Department of Pediatrics, University of Washington, Seattle; 3 Vaccine and Infectious Disease Division, Fred Hutchinson Cancer Center, Seattle, Washington; 4 Amazon, Seattle, Washington; 5 Center for Infectious Disease and Vaccine Research, La Jolla Institute for Immunology, La Jolla, California; 6 St Luke’s Medical Center, Ketchum, Idaho; 7 Division of Infectious Diseases and Global Public Health, Department of Medicine, University of California, San Diego, La Jolla; 8 Department of Emergency Medicine, University of Washington, Seattle; 9 Division of Allergy and Infectious Diseases, Department of Medicine, University of Washington, Seattle

## Abstract

**Importance:**

The US arrival of the Omicron variant led to a rapid increase in SARS-CoV-2 infections. While numerous studies report characteristics of Omicron infections among vaccinated individuals or persons with previous infection, comprehensive data describing infections among adults who are immunologically naive are lacking.

**Objectives:**

To examine COVID-19 acute and postacute clinical outcomes among a well-characterized cohort of unvaccinated and previously uninfected adults who contracted SARS-CoV-2 during the Omicron (BA.1/BA.2) surge, and to compare outcomes with infections that occurred during the Delta wave.

**Design, Setting, and Participants:**

This prospective multisite cohort study included community-dwelling adults undergoing high-resolution symptom and virologic monitoring in 8 US states between June 2021 and September 2022. Unvaccinated adults aged 30 to less than 65 years without an immunological history of SARS-CoV-2 who were at high risk of infection were recruited. Participants were followed for up to 48 weeks, submitting regular COVID-19 symptom surveys and nasal swabs for SARS-CoV-2 polymerase chain reaction (PCR) testing. Data were analyzed from May to October 2022.

**Exposures:**

Omicron (BA.1/BA.2 lineages) vs Delta SARS-CoV-2 infection, defined as a positive PCR test result that occurred during a period when the variant represented at least 50% of circulating SARS-CoV-2 variants in the participant’s geographic region.

**Main Outcomes and Measure(s):**

The main outcomes examined were the prevalence and severity of acute (≤28 days after onset) and postacute (≥5 weeks after onset) symptoms.

**Results:**

Among 274 participants who were immunologically naive (mean [SD] age, 49 [9.7] years; 186 [68%] female; 19 [7%] Hispanic participants; 242 [88%] White participants), 166 (61%) contracted SARS-CoV-2. Of these, 137 infections (83%) occurred during the Omicron-predominant period and 29 infections (17%) occurred during the Delta-predominant period. Asymptomatic infections occurred among 7% (95% CI, 3%-12%) of Omicron-wave infections and 0% (95% CI, 0%-12%) of Delta-wave infections. Health care use among individuals with Omicron-wave infections was 79% (95% CI, 43%-92%) lower relative to individuals with Delta-wave infections (*P* = .001). Compared with individuals infected during the Delta wave, individuals infected during the Omicron wave also experienced a 56% (95% CI, 26%-74%, *P* = .004) relative reduction in the risk of postacute symptoms and a 79% (95% CI, 54%-91%, *P* < .001) relative reduction in the rate of postacute symptoms.

**Conclusions and Relevance:**

These findings suggest that among adults who were previously immunologically naive, few Omicron-wave (BA.1/BA.2) and Delta-wave infections were asymptomatic. Compared with individuals with Delta-wave infections, individuals with Omicron-wave infections were less likely to seek health care and experience postacute symptoms.

## Key Points

**Question:**

What are acute and postacute outcomes among adults previously uninfected and unvaccinated who were infected with SARS-CoV-2 during the Omicron (BA.1/BA.2) wave, and how do these compare with infections during the Delta wave?

**Findings:**

In this cohort study of 274 immunologically naive adults aged 30 to less than 65 years, 61% contracted SARS-CoV-2, with 6% of infections being asymptomatic. Compared with infections during the Delta wave, people infected during the Omicron wave experienced a 79% relative risk reduction in health care use, 56% reduction in the relative risk of postacute symptoms, and 79% reduction in the relative rate of postacute symptoms.

**Meaning:**

These findings suggest that asymptomatic infection may have been relatively rare among immunologically naive adults and that, compared with infections during the Delta wave, individuals infected during the Omicron wave had lower likelihoods of severe illness and postacute symptoms.

## Teaser

This cohort study examines acute and postacute clinical outcomes of SARS-CoV-2 infection during the Delta and Omicron waves among previously immunologically naive adults.

## Introduction

COVID-19 public health policies and control efforts must consider evolving clinical and epidemiological features of disease.^1,2,3^ Since these features can be impacted by SARS-CoV-2 genomic changes, the World Health Organization recommends reevaluation of the clinical course of COVID-19 with the arrival of new SARS-CoV-2 variants.^3^ In December 2021, the B.1.1.529 (Omicron) variant of concern became the predominant SARS-CoV-2 variant circulating in the US, followed by a rapid rise in cases. Between December 2021 and March 2022, the US seroprevalence of infection-induced SARS-CoV-2 antibodies was estimated to have increased by nearly 25%,^4^ with a 35% increase observed among adults who were unvaccinated.^5^

Although numerous studies have found differences in Omicron variant clinical outcomes compared with previous variants,^6,7,8,9,10,11^ these findings are difficult to interpret due to changes in population immunity from natural infection and vaccinations.^12,13,14^ To date, longitudinal, community-based cohort studies examining Omicron infection characteristics have predominantly studied vaccinated cohorts.^7,15^ However, investigating clinical outcomes among immunologically naive populations remains important to understand the consequences of SARS-CoV-2 genomic variation.

In this study, we examined acute COVID-19 and postacute COVID-19 (also called *long COVID* or *post–COVID-19 condition* [PCC]) clinical outcomes during a period of Omicron (BA.1 and BA.2 lineages) variant predominance among a community-based prospective cohort of adults who were immunologically naive with high risk of infection and undergoing high-resolution symptom and virologic surveillance. To identify differences in illness, we compared outcomes with study participants who contracted SARS-CoV-2 during a period of Delta variant predominance.

## Methods

This cohort study was reviewed and approved by the Western Institutional Review Board. All participants provided electronic written informed consent. This study is reported following the Strengthening the Reporting of Observational Studies in Epidemiology (STROBE) reporting guideline.

### Design, Setting, and Participants

The COVID-19 Immune Protection Study (COVIDIPS) is a multisite, prospective cohort study designed to examine SARS-CoV-2 innate and adaptive immune responses among adults who are immunologically naive. To ensure an adequate sample size, participants were recruited using web-based advertisements from targeted geographic locations with high SARS-CoV-2 community transmission and suboptimal COVID-19 vaccination rates in 8 US states (Alabama, Arizona, California, Idaho, Nevada, Oregon, Utah, and Washington). To ensure feasibility of study procedures, recruitment locations must have been within the service region of a national mobile phlebotomy company or in close-proximity to 1 of 2 research sites.

Participants were recruited between March 2021 and February 2022. At enrollment, eligible individuals were aged 30 to less than 65 years, without a previous history of COVID-19 vaccination or SARS-CoV-2 infection, and deemed at high risk for contracting SARS-CoV-2 based on self-reported attitudes and behaviors toward COVID-19 (eMethods in Supplement 1). Participant age was restricted to limit differences in COVID-19 severity associated with age. Similarly, we excluded volunteers who reported any of the following conditions to limit differences in COVID-19 severity due to immune status: current use of immunosuppressive medication, chronic hepatitis B, chronic hepatitis C, HIV, lupus, sarcoidosis, a prior history of SARS-CoV-2 prophylaxis, current pregnancy, or weight less than 110 pounds. Volunteers were screened via an electronic survey in English or Spanish. Eligible individuals were contacted via telephone and invited to participate. Self-reported race and ethnicity data were collected via survey to describe demographics of the cohort and examine potential differences in study outcomes. Race was collected using a multiselect variable with the following categories: American Indian or Alaskan Native, Asian, Black or African American, Native Hawaiian or other Pacific Islander, White, other, and prefer not to say. Ethnicity was collected as a multiple-choice variable with the following answer selections: Hispanic or Latino, not Hispanic or Latino, and prefer not to say.

### Data and Sample Collection

Study data were collected and managed using REDCap (Vanderbilt University), a secure, web-based software platform designed to support data capture for research studies, hosted by the Fred Hutchinson Cancer Center.^16,17^ To confirm immune status at enrollment, participants submitted a blood sample, and SARS-CoV-2 receptor binding domain (RBD) immunoglobin G (IgG) end point titers were quantified. Participants who had either a positive RBD IgG result or a borderline RBD IgG result and positive nucleocapsid IgG result were excluded from the study. Following enrollment, participants completed a baseline questionnaire and began weekly routine procedures for 24 weeks consisting of both an electronic survey to report COVID-19 symptoms and a self-collected nasal swab submitted for SARS-CoV-2 reverse transcription–polymerase chain reaction (RT-PCR) testing (eMethods in Supplement 1). If a participant reported COVID-19 symptoms or had an RT-PCR swab test result positive for SARS-CoV-2 infection, the participant was placed on enhanced procedures consisting of daily electronic symptom surveys and self-collected nasal swabs submitted every other day for up to 14 days. Participants who had a positive SARS-CoV-2 test result and reported symptoms (or did not complete their daily symptom survey) on day 14 of enhanced procedures were sent symptom surveys for an additional 14 days (ie, through day 28 after initiation of enhanced procedures).

After 24 weeks, participants completed an end of study blood draw and questionnaire. Participants were also offered an opportunity to reconsent for an additional 24 weeks of follow-up, consisting of the same study procedures as the previous 24 weeks.

### Exposure Ascertainment

SARS-CoV-2 infection was defined as at least 1 positive RT-PCR result with an infection index date during the time periods that Delta or Omicron (BA.1/BA.2) variants represented the predominant (≥50%; modified in sensitivity analyses to ≥75%) SARS-CoV-2 variant circulating in the participant’s geographic region. Weighted, regional NowCast model estimates from the US Centers for Disease Control and Prevention’s national genomic surveillance system were used to ascertain dates to categorize Omicron-wave (BA.1/BA.2) vs Delta-wave infections (eMethods in Supplement 1).^18^

The infection index date was defined as the first of either the participant’s symptom onset or first SARS-CoV-2–positive RT-PCR swab collection date. Symptom onset dates were determined by participant self-report; where missing, the first date a symptom was reported on surveys was used.

### Outcome Ascertainment

Acute COVID-19 symptoms were defined as symptoms within 28 days of the participant’s symptom onset date, which represents a time period frequently used to demarcate the transition to PCC.^19,20,21^ To minimize misclassification due to nonspecific conditions, a window of 14 days within the collection date of the first positive RT-PCR result was used to examine COVID-19 symptoms; to be considered asymptomatic, participants must have both denied symptoms in all surveys submitted during this time period and submitted at least 1 survey indicating they did not have symptoms 7 or more days after RT-PCR testing to ensure sufficient follow up.^2^ Postacute symptoms were defined as any symptom the participant reported as “related to [their] previous COVID-19 illness” 5 weeks or more after symptom onset (or first positive RT-PCR result for asymptomatic participants). This time period was selected because it was consistent with study procedures (ie, a return to weekly procedures at up to 28 days following enhanced procedures) and common definitions of PCC as symptoms experienced at least 4 weeks after infection.^19,20,21^ In sensitivity analyses, we explored alternate postacute outcome definitions, modifying this time period with and without an upper time limit for reported symptoms, the number of surveys required with reports of postacute symptoms, or postacute symptoms considered. Participants recorded postacute symptoms in routine weekly surveys until the first of either the participant’s study end date or the analyses end date (September 9, 2022).

### Statistical Analysis

Sample sizes for analyses were fixed based on the number of infections observed in our cohort. Risks and 95% CIs of acute asymptomatic infections and postacute symptoms were estimated overall and by variant among participants immunologically naive at the time of infection; relative risks (RRs) and 95% CIs were estimated comparing Omicron-wave infections with Delta-wave infections as the reference group. When RR could not be estimated (due to zero outcomes among the reference group), absolute risk differences and 95% CIs were estimated. To account for differences in postacute symptom follow-up time by variant, rate ratios with 95% CIs were estimated using a generalized estimating equation approach with a Poisson distribution and offset term to account for completed surveys. In these analyses, a robust sandwich covariance estimator with small sample size correction was used to estimate 95% CIs using an exchangeable correlation structure.^22,23^ These models were extended to include multivariable adjustment for participant age (continuous) and gender (binary). In all analyses, a complete case analysis was performed that excluded missing data and 2-tailed α < .05 was used to denote statistical significance.

Descriptive analyses were performed to analyze the prevalence, mean number, and mean severity of acute or postacute symptoms in relation to participant symptom onset. Exploratory multivariable analyses were also conducted to examine the association between acute illness and postacute symptoms (eMethods in Supplement 1).

Baseline and end of study self-rated overall health, memory and concentration, and ability to walk or climb stairs were compared by SARS-CoV-2 variant. For each variable, a change score analysis was performed, comparing the difference in the participant’s end of study response from baseline after adjustment for their baseline score. In primary analyses, Omicron-wave and Delta-wave infections were directly compared; in secondary analyses, each variant was compared with participants who remained SARS-CoV-2 naive at the end of study survey. For inclusion in either analysis, participants with positive SARS-CoV-2 test results must have had at least 5 weeks between their end of study survey and symptom onset (or first positive RT-PCR result date for asymptomatic participants).

All analyses were conducted using RStudio with R statistical software version 4.2.0 (R Project for Statistical Computing). Data were analyzed from May to October 2022.

## Results

During the study period, 274 participants (mean [SD] age, 49 [9.7] years; 186 [68%] female; 19 [7%] Hispanic participants; 242 [88%] White participants) who were immunologically naive were enrolled in the COVIDIPS cohort. Among these participants, 166 (61%) had an RT-PCR test result positive for SARS-CoV-2 during the study period, without a prior history of vaccination or disease; 137 infections (83%) occurred during the Omicron-predominant period and 29 infections (17%) occurred during the Delta-predominant period. Acute symptom data were available for 164 infections (99%), postacute symptom data were available for 150 infections (90%), and end of study survey data at least 5 weeks after infection were available for 133 participants (80%). Demographics of the COVIDIPS cohort are compared by infection status in eTable 4 in Supplement 1, and a study flowchart is provided in eFigure 1 in Supplement 1.

### Acute Symptoms and Health Care Use

Among 164 participants with first-time infections and acute survey data, 9 participants (6% [95% CI, 3%-10%]) did not report symptoms and were classified as asymptomatic. Stratified by variant, 0% (95% CI, 0%-12%) of individuals infected during the Delta wave and 7% (95% CI, 3%-12%) of individuals infected during the Omicron wave were asymptomatic, representing a 7% (95% CI, 3%-11%) absolute risk reduction in symptomatic illness among individuals with Omicron-wave infections compared with individuals with Delta-wave infections (*P* = .002).

**Figure 1.**
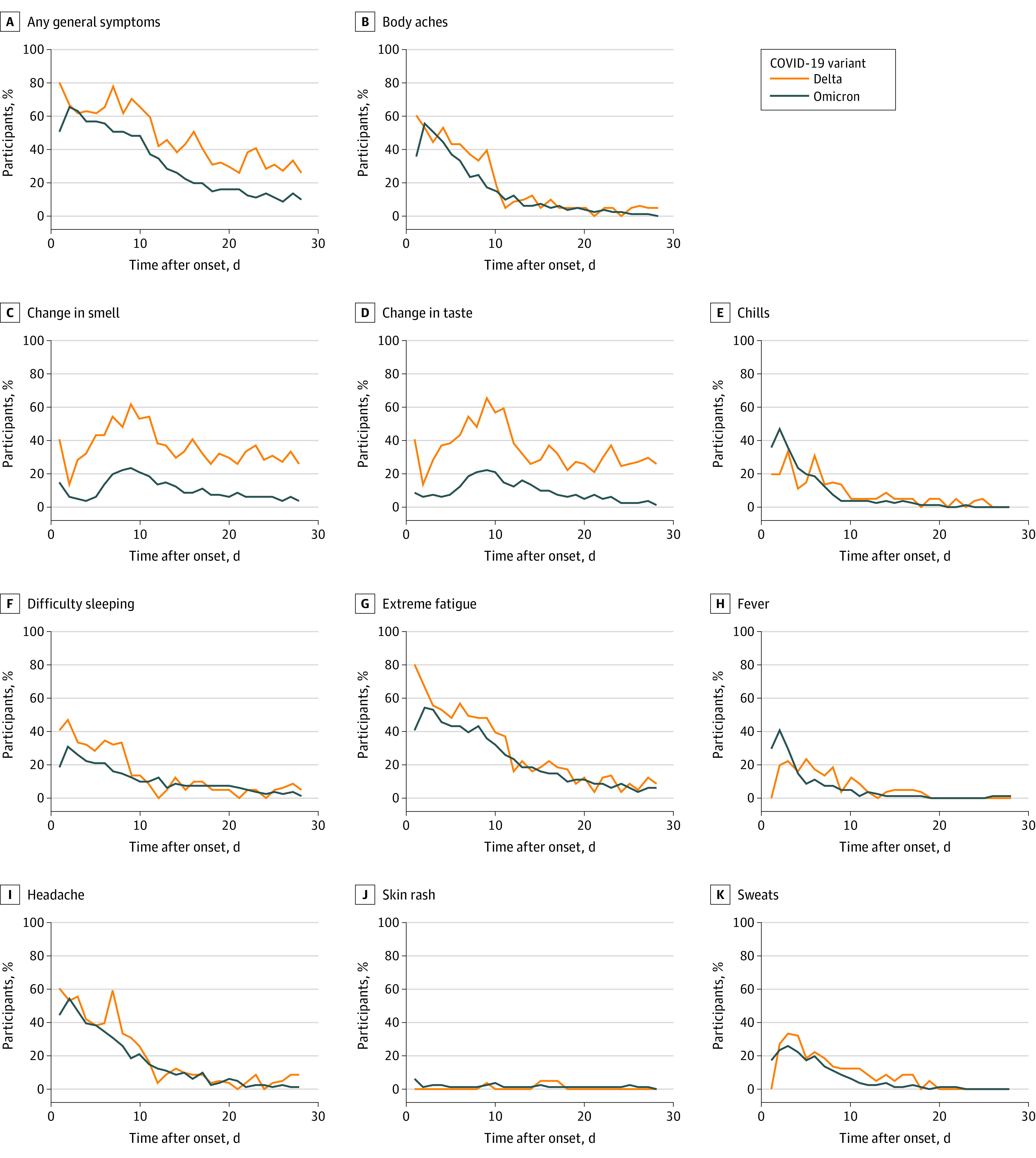
Prevalence of Acute COVID-19 General Symptoms by Variant Percentages were estimated among participants who submitted a survey for that day. Only participants experiencing symptoms are included (n = 155).

**Figure 2.**
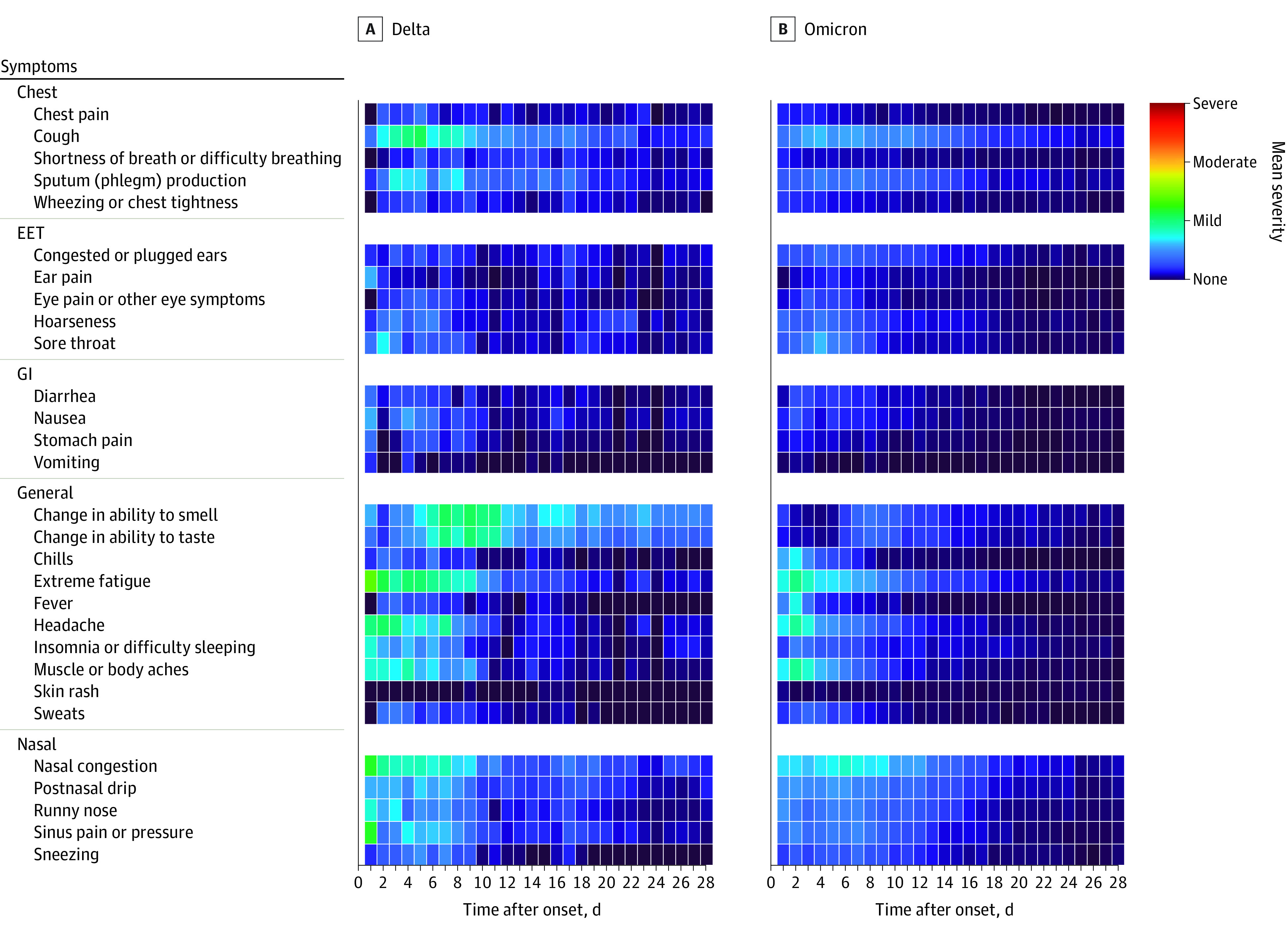
Mean Severity of Acute COVID-19 Symptoms by Variant Percentages were estimated among participants who submitted a survey for that day. Only participants experiencing symptoms are included (n = 155). EET indicates eyes, ears, and throat; GI, gastrointestinal.

Acute symptom prevalence and severity among the 155 symptomatic infections are presented by days since onset in Figure 1 for general symptoms only, eFigure 2 in Supplement 1 for other symptoms, and by severity in Figure 2. The mean daily numbers of acute symptoms are presented in eFigure 3 in Supplement 1. In general, the prevalence of COVID-19 symptoms was similar by variant (Figure 1; eFigure 2 in Supplement 1); however, participants with Delta-wave infections were more likely to report a loss of taste or smell (Figure 1) and rate these symptoms with a higher mean severity (Figure 2).

Overall, 14 participants (9% [95% CI, 5%-14%]) sought health care for acute symptoms, with no participants requiring emergency care or hospitalization. The RR of any health care use was 79% (95% CI, 43%-92%) lower among participants with Omicron-wave infections compared with individuals with Delta-wave infections (risk ratio, 0.21 [95% CI, 0.08-0.57]; *P* = .001). Overall, 9 participants (6% [95% CI, 3%-10%]) received a prescribed medication (eAppendix in Supplement 1), with an 83% (95% CI, 40%-95%) RR reduction among participants with Omicron-wave infections compared with participants with Delta-wave infections (*P* = .002). No differences in acute symptoms, health care use, or medication use were found in sensitivity analyses using modified definitions to classify Omicron-wave and Delta-wave infections (eAppendix in Supplement 1).

### Postacute Symptoms

Among 150 participants with postacute data, 123 participants were infected during the Omicron-predominant period and 27 participants were infected during the Delta-predominant period. Overall, 39 participants (26% [95% CI, 19%-34%]) reported at least 1 symptom during the postacute period. Stratified by variant, 26 participants (21% [95% CI, 14%-29%]) infected during the Omicron period and 13 participants (48% [95% CI, 29%-68%]) infected during the Delta wave reported postacute symptoms, representing a 56% (95% CI, 26%-74%) RR reduction in postacute symptoms among participants infected during the Omicron wave (risk ratio, 0.44 [95% CI, 0.26-0.74] *P* = .004). No differences were found in sensitivity analyses that used modified definitions to classify Omicron-wave and Delta-wave infections (eAppendix in Supplement 1). When analyzed as a rate, 4.9 (95% CI, 3.9-5.9) participants per 100 person-weeks experienced postacute symptoms during the Omicron-predominant period and 29.2 (95% CI, 25.4, 33.1) participants per 100 person-weeks experienced postacute symptoms during the Delta-predominant period (rate ratio, 0.21 [95% CI, 0.09-0.46]; *P* < .001). Similar rate ratio reductions were found after adjustment for participant age and gender. Results from sensitivity analyses demonstrated similar RR reductions for most modified postacute definitions (11 of 13 definitions) (Table). In general, the magnitude of RR reductions among participants with Omicron-wave infections were more pronounced under more stringent postacute definitions; however, risk ratio estimates were imprecise. The 2 definitions examining only cognitive postacute symptoms found no differences between variants.

**Table.** Modified Definitions, Risks, and Risk Ratios (RR) of Postacute Symptoms Comparing Omicron vs Delta Variants^a^

Definition No.	Definition criteria				Risk, % (95% CI)		Risk ratio (95% CI)	*P* value
	Starting wk	Ending wk	Surveys with symptoms required, No.	Symptoms	Omicron (BA.1/BA.2)	Delta (B.1.617.2)		
1^b^	≥5	None	≥1	Any	21 (14-29)	48 (29-68)	0.44 (0.26-0.74)	.004
2	≥5	None	≥1	Cognitive only^c^	12 (7-19)	15 (4-34)	0.82 (0.30-2.29)	.71
3	≥5	None	≥2	Any	13 (8-20)	41 (22-61)	0.32 (0.17-0.61)	<.001
4	≥8	None	≥1	Any	20 (13-28)	44 (25-65)	0.45 (0.26-0.78)	.008
5	≥8	None	≥2	Any	8 (4-14)	37 (19-58)	0.21 (0.09-0.46)	<.001
6	≥12	None	≥1	Any	10 (5-17)	42 (23-63)	0.23 (0.11-0.47)	<.001
7	≥12	None	≥2	Any	4 (1-9)	35 (17-56)	0.11 (0.04-0.33)	<.001
8	≥5	≤24	≥1	Any	20 (14-29)	44 (25-65)	0.46 (0.26-0.79)	.009
9	≥5	≤24	≥1	Cognitive only^c^	11 (6-18)	11 (2-29)	1.02 (0.32-3.32)	.97
10	≥5	≤24	≥2	Any	13 (8-20)	37 (19-58)	0.35 (0.18-0.69)	.003
11	≥8	≤24	≥1	Any	19 (12-27)	41 (22-61)	0.47 (0.26-0.84)	.02
12	≥8	≤24	≥2	Any	8 (4-14)	30 (14-50)	0.26 (0.11-0.62)	.002
13	≥12	≤24	≥1	Any	9 (4-16)	40 (21-61)	0.21 (0.10-0.47)	<.001
14	≥12	≤24	≥2	Any	4 (1-9)	28 (12-49)	0.14 (0.04-0.43)	<.001

^a^ The reference group for all comparisons was individuals infected during the Delta-prominent period.

^b^ Definition 1 was used for primary analyses.

^c^ Cognitive symptoms defined as reports of any of the following symptoms: changes in mood, confusion, extreme fatigue, inability to concentrate, insomnia, or memory lapses.

We examined mean severity of postacute symptoms by variant and individual postacute symptom trajectories in eFigure 4 and eFigure 5 in Supplement 1. To ensure comparability, analyses were restricted to 142 participants (95%) who were unvaccinated and who did not experience SARS-CoV-2 reinfection; 33 participants (22 participants with Omicron-wave infections; 11 participants with Delta-wave infections) reported postacute symptoms. While these analyses represent small numbers of participants, they may suggest differences in postacute symptom severity by strain, with Delta-wave infections reporting higher mean severity and frequency of postacute symptoms.

**Figure 3.**
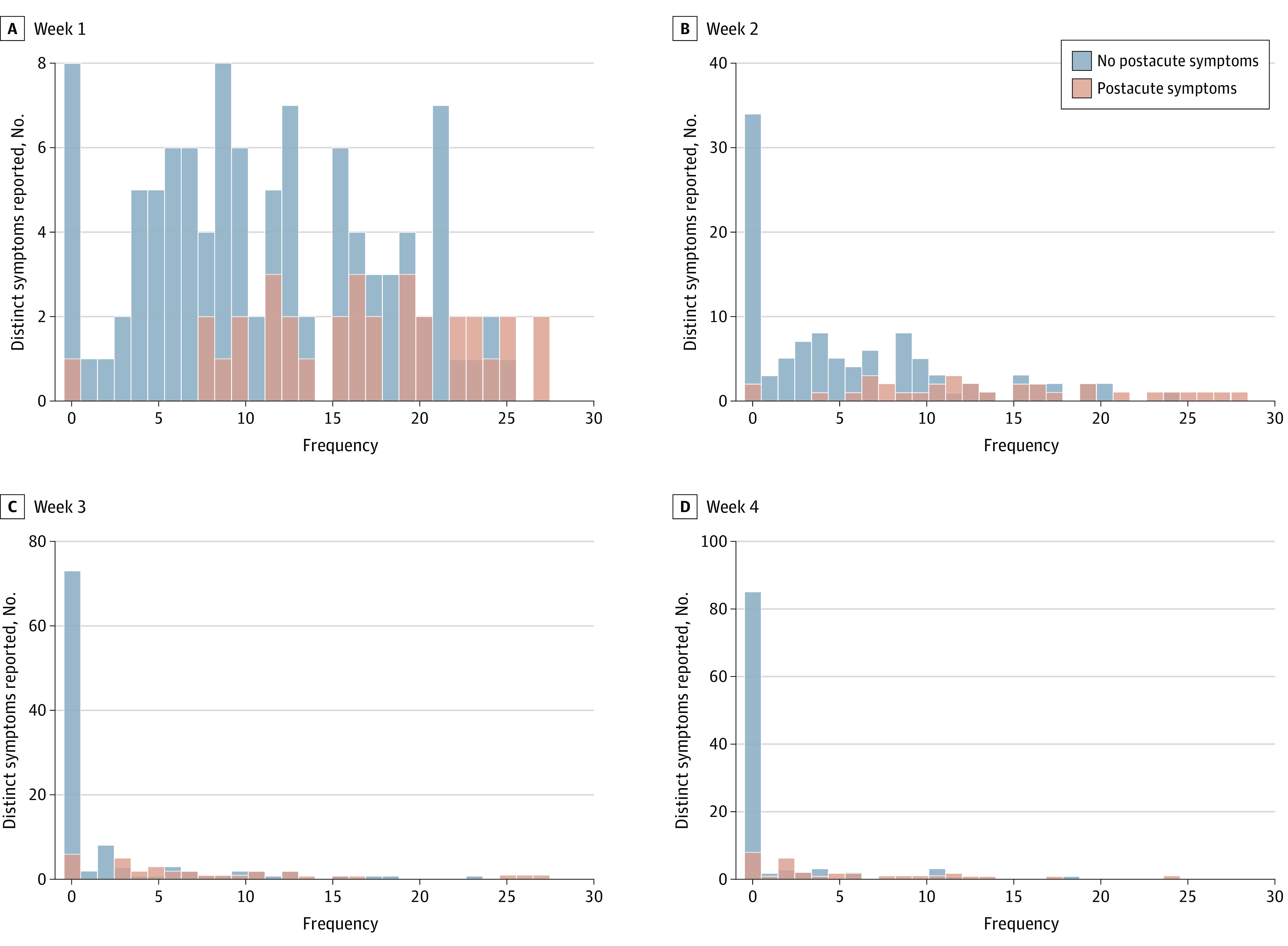
Histogram of Distinct Acute Symptoms Reported by Week and Postacute Symptom Status Only persons who were not vaccinated and had 1 episode of SARS-CoV-2 were included (n = 142).

Given the small number of participants with postacute symptoms, we grouped Delta- and Omicron-wave infections together to examine associations between acute illness and postacute symptoms. Figure 3 and eFigure 6 and eFigure 7 in Supplement 1 illustrate the total number of distinct symptoms, prevalence of acute symptoms, and symptom severity among these participants by postacute status, while results from exploratory multivariable analyses examining the association between acute and postacute outcomes are reported in eTable 5 in Supplement 1. In general, these analyses suggest that increasing acute symptom severity, particularly during the first 2 weeks of acute illness, was associated with higher relative odds and rates of postacute symptoms, and that prevalence of many acute symptoms was greater among participants who experienced postacute symptoms.

### Changes in Baseline and End of Study Self-rated Scores

**Figure 4.**
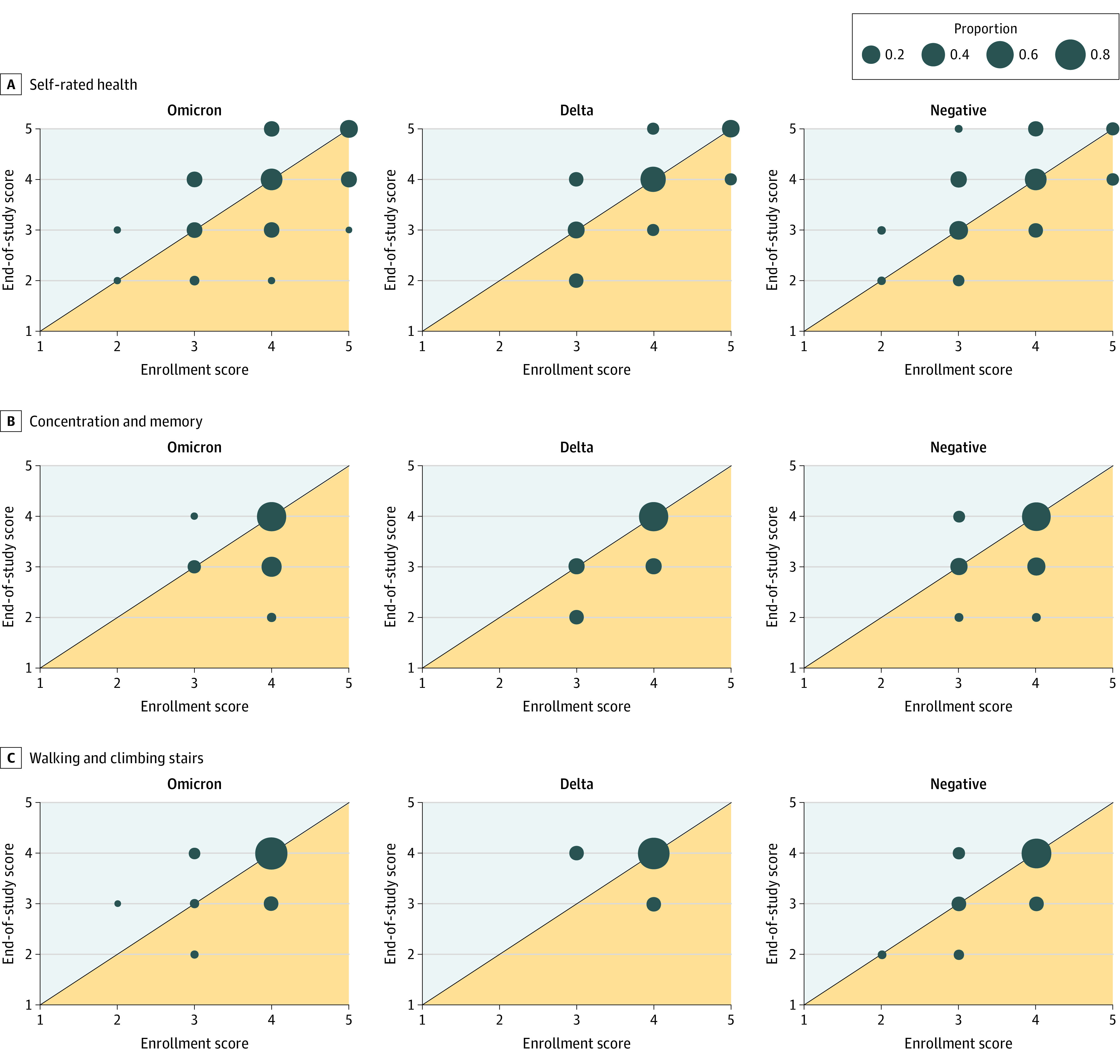
Comparison of Participant Enrollment and End of Study Self-rated Health and Ability Scores At each time point, participants rated their overall health (range, 1-5; higher score indicates better health), concentration & memory (range, 1-4; higher score indicates better abilities), and ability to walk and climb stairs (range, 1-4; higher score indicates better abilities). Dots represent the proportion of participants by variant and SARS-CoV-2 status whose responses align with a given survey pattern. Areas shaded in orange represent a decline in score at the end of study survey, while shaded blue areas represent a positive score gain at the end of study.

Among 133 participants (23 participants with Delta-wave infections; 110 participants with Omicron-wave infections) with end of study data at least 5 weeks after symptom onset, no changes were detected by variant in self-rated measures for overall health, memory or concentration, or physical abilities between enrollment and end of study surveys (Figure 4). These results were similar for primary analyses comparing variants directly, and secondary analyses comparing participants infected during each variant period with 90 participants who remained immunologically naive at their end of study survey (Figure 4).

## Discussion

In this cohort study, we describe COVID-19 acute and postacute outcomes in a well-characterized, community-based, prospective cohort of adults who were unvaccinated and previously uninfected with high risk of contracting SARS-CoV-2 infection based on self-reported behaviors and attitudes toward COVID-19. Our data demonstrate that SARS-CoV-2 infections during the Omicron (BA.1/BA.2) period were associated with significantly lower absolute risks of acute symptomatic infections and significant relative reductions in health care use for acute illness and postacute symptoms at 5 or more weeks compared with Delta-wave infections. While several studies have also found lower acute severity of Omicron-wave infections compared with Delta-wave infections, these studies have included predominantly vaccinated populations.^7,11^ To our knowledge, this is the first study to characterize both acute and postacute symptoms in a cohort of individuals who were immunologically naive in the era of widespread vaccination and increased SARS-CoV-2 seroprevalence using prospective surveillance.

Defining the spectrum of COVID-19 severity for SARS-CoV-2 variants remains important to elucidate the clinical consequences of SARS-CoV-2 genomic variation.^1,2,3^ For these purposes, exploring outcomes among persons who were unvaccinated and previously uninfected may be of particular significance for understanding the natural course of infection in the absence of preexisting host immunity, facilitating comparisons between variants, and identifying outcomes of COVID-19 among nonnaive populations, should immunity wane to levels that do not influence illness severity.

Importantly, as COVID-19 hospitalization and mortality rates have fallen, other end points, such as symptom prevalence and severity, have become critical to characterize different variants. In this study, we evaluated epidemiological characteristics of mild COVID-19 using a prospective cohort design with frequent and high-resolution symptom and virologic surveillance. With these rigorous methods, we were able to provide key insights regarding the prevalence of asymptomatic SARS-CoV-2 infections, which is difficult to ascertain using cross-sectional designs that cannot distinguish between presymptomatic and asymptomatic infection^2^ or cohorts recruited at a health care setting, which may be increasingly unrepresentative of mild disease in an era of pervasive at-home SARS-CoV-2 diagnostics.^24^ Overall, we found that the prevalence of asymptomatic infections was only 6% among all SARS-CoV-2 infections, with a significantly higher absolute prevalence during the Omicron-predominant period (7%) vs the Delta-predominant period (0%). Our estimates of asymptomatic Omicron-wave infections were lower compared with a 2022 systematic review and meta-analysis^25^ that also found a significantly higher proportion of asymptomatic infections among Omicron-wave (25%) vs Delta-wave (8%) infections. This apparent difference may reflect characteristics of our study cohort of previously immunologically naive adults aged 30 to 64 years, since both factors are also associated with a lower prevalence of asymptomatic disease.^25^

Our prospective design also enabled us to estimate risks of postacute COVID-19 symptoms among a fully enumerated cohort of individuals with SARS-CoV-2 infections, which may be more accurate than designs that recruit participants at health care settings or after the development of postacute symptoms.^1^ We found that approximately one-quarter of participants (26%) experienced at least 1 postacute symptom 5 or more weeks after their infection. Our study is also among few to date that compared the risks of postacute COVID-19 symptoms by variant, and the only study to our knowledge that compared postacute risks among individuals who were unvaccinated. We found that risks of postacute symptoms at 5 or more weeks differed significantly by variant, with approximately one-fifth of participants with Omicron-wave infections (21%) vs nearly one-half of participants with Delta-wave infections (48%) experiencing postacute symptoms, representing a relative risk reduction of more than 50% or a rate ratio reduction of 79% among Omicron-wave infections. These findings remained robust under more stringent definitions of postacute symptoms examining symptoms at 8 or more and 12 or more weeks. Our results were also similar to results from a 2022 study^26^ of individuals who were vaccinated that found 59% to 77% reductions in the odds of postacute symptoms among Omicron-wave infections compared with Delta-wave infections.

This study has several strengths. Our recruitment methods and prospective design enabled us to ascertain participants’ baseline immune status with greater certainty than studies using administrative data. Our study procedures also featured frequent symptom and virologic surveillance with little missing data and high retention of study participants, allowing us to characterize outcomes with greater accuracy than cohorts ascertained following infection. Furthermore, the distribution of SARS-CoV-2 infections across Delta and Omicron time periods permitted us to compare and detect significant differences in variant outcomes using the same study procedures. Additionally, during an era of widespread infections and vaccinations, our study population of adults who were immunologically naive also represents a unique cohort of individuals who, at the time of the study, comprised a significant proportion of the US population yet remain underrepresented in scientific research.

### Limitations

Our study has several limitations. We did not sequence viral samples and used US regional prevalence data to classify variants for our SARS-CoV-2 infections. While this approach is similar to methods in previous studies,^7,26^ it is possible that variants were misclassified. However, in sensitivity analyses using more stringent definitions to classify SARS-CoV-2 variants, we found similar results for our primary outcomes. Due to the prospective nature of our study and our unique study cohort, our sample size was also smaller compared with studies using administrative data. Furthermore, since our study relied on participant self-report, it is possible that study participants were more likely to report COVID-19 symptoms than nonparticipants for reasons related to study participation (volunteer bias), or because of enhanced symptom surveillance due to study procedures (surveillance bias). While these potential biases may cause us to overestimate the percentage of infections with symptoms, the biases are unlikely to affect observed differences in acute or postacute symptoms by SARS-CoV-2 variant.

## Conclusions

In this cohort study, we found that among a community-based cohort of adults who were immunologically naive aged 30 to less than 65 years, SARS-CoV-2 infections during Omicron (BA.1/BA.2)- and Delta-predominant periods were associated with few asymptomatic cases of COVID-19, and that participants with Omicron-wave infections were less likely to seek health care for acute symptoms and less likely to experience postacute symptoms compared with individuals with Delta-wave infections. Continued study and comparisons of symptomatic illness from SARS-CoV-2 variants are critical to understand the implications of SARS-CoV-2 genomic variation and must be considered with regard to host immune status.

## Notes

Supplement 1.
eMethods.
**eAppendix.** Supplemental Results **eTable 1.** Calendar Dates of SARS-CoV-2 Variant Circulation by HHS Region for Primary Analyses **eTable 2.** Calendar Dates of SARS-CoV-2 Variant Circulation by HHS Region for Sensitivity Analyses **eTable 3.** Acute Symptoms by Symptom Category **eTable 4.** Selected Demographic and Other Characteristics Among the COVIDIPS Cohort by SARS-CoV-2 Status **eTable 5.** Results From Multivariable Regressions Examining the Association of Acute COVID-19 Symptoms With Postacute Symptoms **eFigure 1.** Flow Diagram Detailing Enrollment and Outcomes in the COVIDIPS Cohort **eFigure 2.** Prevalence of Acute COVID-19 Symptoms, Excluding General Symptoms, by Variant **eFigure 3.** Mean Number of Acute Symptoms by Day and Variant **eFigure 4.** Mean Severity of Postacute COVID-19 Symptoms by Variant **eFigure 5.** Trajectory of Postacute COVID-19 Symptoms by Variant **eFigure 6.** Prevalence of Acute COVID-19 Symptoms by Postacute Status **eFigure 7.** Mean Severity of Acute Symptoms by Day and Postacute Status

Supplement 2.
Data Sharing Statement
