# Supplementary material for: Acute and Postacute COVID-19 Outcomes Among Immunologically Naive Adults During Delta vs Omicron Waves
Source: JAMA Netw Open. 2023 Feb 28;6(2):e231181. doi: 10.1001/jamanetworkopen.2023.1181 (PMC9975921; doi:10.1001/jamanetworkopen.2023.1181)
Supplement: Supplement 1. — eMethods. eAppendix. Supplemental Results eTable 1. Calendar Dates of SARS-CoV-2 Variant Circulation by HHS Region for Primary Analyses eTable 2. Calendar Dates of SARS-CoV-2 Variant Circulation by HHS Region for Sensitivity Analyses eTable 3. Acute Symptoms by Symptom Category eTable 4. Selected Demographic and Other Characteristics Among the COVIDIPS Cohort by SARS-CoV-2 Status eTable 5. Results From Multivariable Regressions Examining the Association of Acute COVID-19 Symptoms With Postacute Symptoms eFigure 1. Flow Diagram Detailing Enrollment and Outcomes in the COVIDIPS Cohort eFigure 2. Prevalence of Acute COVID-19 Symptoms, Excluding General Symptoms, by Variant eFigure 3. Mean Number of Acute Symptoms by Day and Variant eFigure 4. Mean Severity of Postacute COVID-19 Symptoms by Variant eFigure 5. Trajectory of Postacute COVID-19 Symptoms by Variant eFigure 6. Prevalence of Acute COVID-19 Symptoms by Postacute Status eFigure 7. Mean Severity of Acute Symptoms by Day and Postacute Status [file jamanetwopen-e231181-s001.pdf]

Supplemental Online Content

Doll MK, Waghmare A, Heit A, et al. Acute and postacute COVID-19 outcomes among immunologically naive adults during Delta vs Omicron waves. *JAMA Netw Open*. 2023;6(2):e231181. doi:10.1001/jamanetworkopen.2023.1181

eMethods.

eAppendix. Supplemental Results

eTable 1. Calendar Dates of SARS-CoV-2 Variant Circulation by HHS Region for Primary Analyses

eTable 2. Calendar Dates of SARS-CoV-2 Variant Circulation by HHS Region for Sensitivity Analyses

eTable 3. Acute Symptoms by Symptom Category

eTable 4. Selected Demographic and Other Characteristics Among the COVIDIPS Cohort by SARS-CoV-2 Status

eTable 5. Results From Multivariable Regressions Examining the Association of Acute COVID-19 Symptoms With Postacute Symptoms

eFigure 1. Flow Diagram Detailing Enrollment and Outcomes in the COVIDIPS Cohort

eFigure 2. Prevalence of Acute COVID-19 Symptoms, Excluding General Symptoms, by Variant

eFigure 3. Mean Number of Acute Symptoms by Day and Variant

eFigure 4. Mean Severity of Postacute COVID-19 Symptoms by Variant

eFigure 5. Trajectory of Postacute COVID-19 Symptoms by Variant

eFigure 6. Prevalence of Acute COVID-19 Symptoms by Postacute Status

eFigure 7. Mean Severity of Acute Symptoms by Day and Postacute Status

This supplemental material has been provided by the authors to give readers additional information about their work.

© 2023 Doll MK et al. *JAMA Network Open.*

eMethods.

The following appendix provides additional information regarding the CovidIPS study procedures and statistical methods.

Participant eligibility – ascertainment of high risk status

To ensure an adequate sample size of SARS-CoV-2 infections, we developed a scoring system to measure a volunteer's theoretical risk of contracting SARS-CoV-2. This risk was based upon the following 3 multiple choice survey questions included in the electronic screening questionnaire:

1. How concerned are you about coronavirus (COVID-19)?
2. In an average week, approximately how many people do you have contact with when you are indoors, and NOT wearing a face mask? *Please do not include people who live in your home.*
3. Do you trust that the coronavirus (COVID-19) vaccines are safe?

To be eligible for study participation, volunteers must have received a high-risk score above a pre-determined threshold. Where data from question 3 was not available for a participant, a modified threshold score was applied to ascertain study eligibility.

Nasal swab collection

Participants used foam swabs (Custom Swab Puritan Medical Ref #25-18051-PFSC2ARROW) to self-collect a nasal specimen from the mid-nasal space.[27] Participants were provided written instructions as well as a video link demonstrating sample collection. Participants placed the swab into one nostril and rotated the swabs for 5 rotations. The same swab was then placed in the other nostril also rotated 5 times. The swab was then placed dry into a conical vial and shipped to the lab by the participant. Weekly surveillance swabs were shipped via USPS Priority Mail (1 – 3 days) whereas swabs collected as part of enhanced procedures were shipped via FedEx (week 1 collections: FedEx Priority Overnight; week 2 collections: FedEx 2 Day (3 swabs shipped together at the end of the 2nd week).

© 2023 Doll MK et al. *JAMA Network Open.*

Nasal swab processing

Upon arrival to the laboratory (Atlas Genomics, Seattle, WA), 2mL of PBS was added to each sample tube and vortexed. Samples were either extracted individually or as pooled samples (Pool of 4: 100uL each sample; Pool of 3: 133uL each sample; Pool of 2: 200uL each sample). RNA extraction was performed using the KingFisher™ Flex Magnetic Particle Processor with 96 Deep-Well Head. 5ul Proteinase K was added to each well along with 200ul of sample. 275ul Binding Bead mix (per well: 265ul Binding Solution, 10ul Total Nucleic Acis Magnetic Beads) was then added followed by 5 μL of MS2 Phage Control. The “MV_Flex” run was used, consisting of a Wash Buffer step, 2 washes with 80% ethanol, and an elution buffer step. The elution plate was either placed on ice for immediate use in real-time RT-PCR or stored at -10 to -30°C freezer for short term storage (1 week).

RT-PCR testing

RT-PCR was performed at a diagnostic laboratory (Atlas Genomics Laboratories) based in Seattle, Washington, USA, using the Applied Biosystems™ QuantStudio™ 7 Flex Real-Time PCR Instrument with the TaqPath COVID-19 Combo Kit (Thermo Fisher Scientific).[28]

SARS-CoV-2 variant exposure classification

We classified SARS-CoV-2 infections as Omicron (BA.1/BA.2 lineages) or Delta variants based upon the participant’s infection index date and the dates these variants represented ≥50% (or ≥75 in sensitivity analyses) of SARS-CoV-2 variants circulating in the participant’s U.S. Department of Health and Human Services (HHS) geographic region. Participant HHS geographic region was classified according to the mailing address where the participant received their study supplies.

To identify these SARS-CoV-2 variant thresholds, we used weighted, HHS regional Nowcast model estimates obtained from the U.S. Centers for Disease Control and Prevention’s (CDC) national genomic surveillance system.[18] Calendar dates of SARS-CoV-2 variant circulation by HHS region used for primary and sensitivity analyses are provided in eTables 1 and 2, respectively.

Descriptive analyses examining acute and postacute symptoms

For each time unit analyzed in figures, a complete case analysis was performed using data from symptomatic participants who submitted a survey for that time unit, excluding participants with missing data. Participants who reported no symptoms on day 14 were presumed to remain symptom free on days 15-28 following the start of their enhanced procedures. Sensitivity analyses were conducted to exclude infections where symptom onset date may have been misclassified because it was not reported by the participant or observed across surveys.

Exploratory analyses examining the relationship between acute and postacute symptoms

We examined the relationship between acute COVID-19 illness and postacute symptoms in multivariable analyses using four different modeling approaches to characterize acute and postacute COVID-19 illness. In these exploratory analyses, we adjusted all models for participant age (continuous, centered at 30 years) and gender (binary), and implemented the following specifications of acute and postacute symptoms in separate models:

Acute COVID-19 illness: Since symptoms of acute COVID-19 vary over the course of illness, we created summary variables to describe acute symptoms for each week of the 4-week duration of the acute illness period. Separate models were then run for weeks 1 to 4 to examine the relationships of distinct weekly patterns with the development of postacute outcomes. In these analyses, weekly acute COVID-19 symptoms were characterized in two different ways. First, in base models, we expressed weekly acute COVID-19 symptoms as two variables, indicating the: (i) presence/absence of weekly symptoms as a binary variable, and the (ii) total number of distinct weekly acute symptoms experienced to measure symptom severity as a continuous variable. Results of base models informed the approach to characterize acute symptoms in the subsequent models. Where the presence/absence of weekly symptoms was significant ($\alpha < 0.05$), but the total number of symptoms experienced (severity) was not, models were run to explore the presence/absence of 5 specific categories of acute COVID-19 symptoms (eTable 3), using data only from persons who experienced symptomatic illness during that week. Where the presence/absence of weekly symptoms was non-significant, but the

© 2023 Doll MK et al. *JAMA Network Open.*

total number of symptoms (severity) was significant ($\alpha < 0.05$), the 5 categories of symptoms were entered into the model in addition to the term for the total number of symptoms (severity). Where neither term was significant, the 5 specific categories of acute COVID-19 symptoms were entered, and data from all persons (regardless of symptomatic illness) were explored.

Postacute symptoms. In separate analyses, postacute symptoms ≥5-weeks following onset were examined as either: (i) a dichotomous outcome, representing the presence or absence of symptoms at ≥5-weeks post-onset, or a (ii) a count of the number of study weeks that a participant experienced postacute symptoms ≥5-weeks following onset. Where postacute symptoms were expressed as a binary variable (i), we implemented logistic regressions using a generalized linear model (GLM) approach to examine the odds of postacute symptoms ≥5-weeks following onset. Where postacute symptoms were expressed as a count, we implemented a generalized estimating equation (GEE) approach with a Poisson distribution and offset term to examine rates of weekly postacute symptoms.

© 2023 Doll MK et al. *JAMA Network Open.*

eAppendix. Supplemental Results

Prescription medication use reported for acute COVID-19 illness

Among participants who had acute symptom data and were infected with Omicron, 4 participants (3.0%, 95% CI: 0.8%, 7.4%) reported receiving a medication for their illness. Medications reported were an antibiotic (n=1), an oral steroid (n=1), ivermectin (n=1), and oseltamivir (n=1). Among participants who had acute symptom data and were infected with Delta, 5 participants (17.2%, 5.8%, 35.8%) reported receiving a medication. Medications reported were an antibiotic (n=2), ivermectin (n=2), and an antibody infusion (n=1).

Sensitivity analyses to examine modified definitions of Omicron and Delta infections

When modified definitions to classify SARS-CoV-2 strains were implemented (eTable2), no meaningful or significant differences in primary analyses were found. Using modified Omicron and Delta definitions, there was a 5.9% (95% CI: 1.7%, 10.2%, P =0.006) absolute risk reduction in symptomatic illness among Omicron compared with Delta infections. The RR for seeking any healthcare was 88% (95% CI: 62%, 96%; P<0.001) lower among Omicron relative to Delta infections, and an 87% (95% CI: 50%, 97%, P<0.001) RR reduction in receipt of a prescription medication was observed among Omicron relative to Delta infections. For postacute outcomes, we estimated a 58.2% (95% CI: 28.2%, 75.6%; P=0.003) RR reduction in postacute symptoms among Omicron participants.

Potential misclassification of symptom onset date

Among participants with acute data (n=164), 14 (2 Delta & 12 Omicron) participants had potentially misclassified symptom onset dates. Exclusion of these cases from analyses which examined the time since symptom onset did not reveal meaningful differences in study results (data not shown).

Exploratory analyses examining the relationship between acute and postacute symptoms

Results from multivariable analyses exploring the relationship between acute and postacute symptoms are reported in the eTable 5. In general, we observed different patterns in acute weeks 1 and 2 in comparison with acute weeks 3 and 4. Specifically, we

© 2023 Doll MK et al. *JAMA Network Open.*

found that the total number of symptoms (severity) in acute weeks 1 and 2 was significantly associated with an increase in the odds/rates of postacute symptoms. However, in weeks 3 and 4, this pattern was not apparent. For these weeks, the weekly presence/absence of all acute symptoms was instead significantly associated with an increase in the odds/rates of postacute symptoms. Furthermore, in secondary models with symptom categories, we observed that the presence of chest symptoms in weeks 1 or 2 was (in most cases) significantly/borderline significantly associated with a 4 to 8 times increase in the odds/rates of postacute symptoms. This was in contrast to weeks 3 or 4, where the presence of chest symptoms was non-significant, and instead, the presence of general symptoms was (in some cases) significantly/borderline significantly associated with an increase in the odds/rates of postacute symptoms. While models comparing symptom categories were largely imprecise, point estimates and trends in results from logistic GLM and Poisson GEE regressions were similar.

© 2023 Doll MK et al. *JAMA Network Open.*

eTable 1. Calendar Dates of SARS-CoV-2 Variant Circulation by HHS Region for Primary Analyses

HHS Region	Participant state address	Delta variant (B.1.617.2) Start Date (≥50%; inclusive)	Omicron (BA.1/BA.2)* Start Date (≥50%; inclusive)	Omicron (BA.1/BA.2)* End Date (≥50%; inclusive)
**4**	AL	6/27/2021	12/12/2021	6/18/2022
**8**	UT	6/6/2021	12/19/2021	6/18/2022
**9**	AZ, CA, NV	6/20/2021	12/19/2021	6/18/2022
**10**	ID, OR, WA	6/27/2021	12/19/2021	6/18/2022

**Includes*: B.1.1.529, BA.1.1, BA.2, BA.2.12.1 lineages

© 2023 Doll MK et al. *JAMA Network Open.*

eTable 2. Calendar Dates of SARS-CoV-2 Variant Circulation by HHS Region for Sensitivity Analyses

HHS Region	Participant state address	Delta variant (B.1.617.2) Start Date (≥75%; inclusive)	Delta variant (B.1.617.2) End Date (≥75%; inclusive)	Omicron (BA.1/BA.2)* Start Date (≥75%; inclusive)	Omicron (BA.1/BA.2)* End Date (≥75%; inclusive)
**4**	AL	7/4//2021	12/11/2021	12/19/2021	6/4/2022
**8**	UT	6/20/2021	12/18/2021	12/26/2021	6/4/2022
**9**	AZ, CA, NV	7/4/2021	12/11/2021	12/19/2021	6/4/2022
**10**	ID, OR, WA	7/11/2021	12/11/2021	12/26/2021	6/4/2022

**Includes*: B.1.1.529, BA.1.1, BA.2, BA.2.12.1 lineages

© 2023 Doll MK et al. *JAMA Network Open.*

eTable 3. Acute Symptoms by Symptom Category

Category	Symptom
**Nasal**	Nasal congestion
	Post-nasal drip
	Runny nose
	Sinus pain or pressure
	Sneezing
**Chest**	Chest pain
	Cough
	Shortness of breath or difficulty breathing
	Sputum (phlegm) production
	Wheezing or chest tightness
**General**	Change in ability to smell
	Change in ability to taste
	Chills
	Extreme fatigue (tiredness)
	Fever
	Headache
	Insomnia or difficulty sleeping
	Muscle or body aches
	Skin rash
	Sweats
**Eyes, Ears, & Throat (EET)**	Congested or plugged ears
	Ear pain
	Eye pain or other eye symptoms
	Hoarseness
	Sore Throat
**Gastrointestinal (GI)**	Diarrhea
	Nausea
	Stomach pain
	Vomiting

© 2023 Doll MK et al. *JAMA Network Open.*

eTable 4. Selected Demographic and Other Characteristics Among the COVIDIPS Cohort by SARS-CoV-2 Status

Note SARS-CoV-2 negatives represent participants who contributed person-time to the study, and did not contract SARS-CoV-2 while immunologically naïve

**Demographic Variable or Characteristic**	**SARS-CoV-2 Infection: Omicron period (N = 137)**		**SARS-CoV-2 Infection: Delta period (N = 29)**		**SARS-CoV-2 Negatives (N = 108)**	
	**N**	**% (95% CI)**	**N**	**% (95% CI)**	**N**	**% (95% CI)**
**Gender:** Female	95	69 (61, 77)	20	69 (52, 86)	71	66 (57, 75)
**Age (years):** Mean	48	(47, 50)	46	(42, 49)	51	(49, 53)
**Race (self-report)**						
White	127	93 (88, 97)	25	86 (74, 99)	90	83 (76, 90)
Another+	10	7 (3, 12)	4	14 (1, 26)	18	17 (10, 24)
**Ethnicity (self-report)***						
Not Hispanic or Latino	122	89 (85, 94)	26	90 (83, 100)	97	90 (85, 95)
Hispanic or Latino	10	7 (3, 12)	2	7 (0, 18)	7	7 (2, 12)
**Education***						
High school or equivalent (GED)	44	32 (23, 40)	2	7 (0, 26)	29	27 (18, 38)
Associate or technical degree	38	28 (19, 37)	8	28 (10, 47)	33	31 (22, 42)
Bachelor's degree	35	26 (17, 35)	13	44 (28, 64)	29	26 (18, 38)
Graduate degree	19	14 (5, 22)	6	21 (3, 40)	15	14 (5, 25)
**Employment Status**						
Not employed	26	19 (10, 28)	6	21 (3, 41)	30	28 (19, 38)
Part-time	49	36 (27, 45)	12	41 (24, 61)	21	19 (10, 30)
Full time	62	45 (37, 54)	11	38 (21, 58)	57	53 (44, 63)
**Occupation**						
Agriculture, construction, transportation, and manufacturing	19	17 (9, 27)	2	9 (0, 31)	15	19 (9, 31)
Arts, entertainment, recreation, retail and wholesale trade	14	12 (7, 29)	2	9 (0, 31)	5	7 (0, 18)
Finance, information, Professional, scientific, management, and public administration	17	16 (11, 32)	2	9 (0, 31)	14	18 (8, 30)
Educational services and social assistance	11	10 (4, 25)	3	13 (0, 35)	4	5 (0, 17)
Health care	20	18 (10, 28)	8	34 (17, 57)	18	23 (13, 35)
Others	30	27 (16, 34)	6	26 (9, 48)	22	28 (18, 403
**Household composition**						
Living alone	14	10 (2, 18)	1	3 (0, 24)	19	18 (8, 28)
2 persons	25	19 (10 27)	5	17 (3, 38)	36	32 (24, 43)
3 persons	22	16 (8, 24)	6	21 (7, 41)	20	19 (9, 29)
4+ persons	76	55 (47, 64)	17	59 (45, 79)	33	31 (22, 42)
**Underlying conditions at baseline**						
Anxiety	21	15 (9, 21)	3	10 (0, 21)	22	21 (13, 28)
Asthma	10	7 (3, 11)	4	14 (1, 26)	11	10 (5, 16)
Chronic obstructive pulmonary diseases (COPD) or heart problems	2	1 (0, 3)	0	0	9	9 (3, 14)
Depression	8	6 (2, 10)	2	7 (0, 16)	10	9 (4, 15)
Diabetes	9	7 (2, 11)	2	7 (0, 16)	8	8 (2, 12)
High blood pressure	17	12 (7, 18)	3	10 (0, 21)	18	17 (10, 24)
Post-traumatic stress disorder	5	4 (0, 7)	0	0	5	5 (0, 9)
Thyroid conditions	11	8 (3, 12)	3	10 (0, 21)	12	11 (5, 17)
Other medical conditions	12	9 (4, 13)	5	17 (3, 31)	18	17 (10, 24)
**Masking behavior**						
Always	12	9 (0, 17)	3	10 (0, 28)	18	17 (7, 27)
Mostly	36	26 (17, 35)	4	14 (0, 31)	29	27 (18, 37)
Sometimes	42	30 (22, 40)	12	42 (24, 59)	36	33 (24, 43)
Rarely	39	29 (20, 37)	5	17 (0, 35)	19	17 (8, 28)
Never	8	6 (0, 14)	5	17 (0, 36)	6	6 (0, 16)
**Attends indoor social gatherings**						
Never	12	9 (0, 18)	2	7 (0, 27)	19	18 (8, 28)
Sometimes (≥1x per month)	53	39 (32, 49)	9	31 (17, 51)	49	44 (36, 56)
Frequently (≥1x per week)	72	52 (44, 61)	18	62 (48, 82)	40	38 (28, 48)

+defined as another category(-ies) of race besides White alone; *excludes "prefer not to say" category, does not sum to total

© 2023 Doll MK et al. *JAMA Network Open.*

eTable 5. Results From Multivariable Regressions Examining the Association of Acute COVID-19 Symptoms With Postacute Symptoms

All analyses are adjusted for gender and age.

Model	Variable	Week 1			Week 2			Week 3			Week 4		
**Logistic GLM**		**OR**	**95% CI**	**P-value**	**OR**	**95% CI**	**P-value**	**OR**	**95% CI**	**P-value**	**OR**	**95% CI**	**P-value**
1	Any symptoms	0.4	(0.0, 8.9)	0.459	1.2	(0.2, 9.1)	0.864	6.1	(1.8, 21.9)	0.004*	12.4	(3.5, 46.3)	<0.001*
1	Symptom severity	1.2	(1.1, 1.3)	<0.001*	1.2	(1.1, 1.3)	<0.001*	1.1	(1.0, 1.2)	0.164	1.0	(0.9, 1.2)	0.495
2	Symptom severity	1.2	(1.1, 1.4)	0.008*	1.2	(1.1, 1.4)	0.005*	----			----		
2	Nasal symptoms	0.7	(0.1, 4.2)	0.645	0.5	(0.1, 3.6)	0.503	1.4	(0.2, 10.0)	0.748	0.4	(0.1, 2.8)	0.397
2	Chest symptoms	5.8	(1.3, 34.2)	0.031*	8.2	(1.7, 53.6)	0.014*	2.6	(0.6, 12.7)	0.209	2.5	(0.4, 20.5)	0.361
2	General symptoms	1.3	(0.2, 10.5)	0.793	0.8	(0.2, 4.2)	0.778	5.3	(1.3, 27.2)	0.030*	4.6	(1.0, 23.5)	0.051
2	EET symptoms	0.3	(0.1, 1.3)	0.111	0.4	(0.1, 1.4)	0.160	0.8	(0.2, 3.1)	0.725	1.3	(0.2, 7.3)	0.765
2	GI symptoms	0.3	(0.1, 1.1)	0.073	0.5	(0.1, 1.7)	0.268	0.3	(0.1, 1.6)	0.170	0.2	(0.0, 1.5)	0.130
**Poisson GEE**		**RR**	**95% CI**	**P-value**	**RR**	**95% CI**	**P-value**	**RR**	**95% CI**	**P-value**	**RR**	**95% CI**	**P-value**
3	Any symptoms	1.0	(0.1, 14.8)	0.982	2.1	(0.4, 10.3)	0.373	4.0	(1.2, 14.1)	0.028*	7.9	(2.6, 24.5)	0.001*
3	Symptom severity	1.1	(1.1, 1.2)	0.001*	1.1	(1.1, 1.2)	<0.001*	1.1	(1.0, 1.1)	0.107	1.1	(0.9, 1.2)	0.232
4	Symptom severity	1.1	(1.0, 1.3)	0.092	1.1	(1.0, 1.3)	0.059	----			----		
4	Nasal symptoms	1.2	(0.1, 9.2)	0.88	1.5	(0.3, 7.5)	0.613	0.5	(0.1, 2.5)	0.323	0.5	(0.2, 1.5)	0.169
4	Chest symptoms	4.2	(1.0, 17.5)	0.052	4.0	(0.7, 23.5)	0.126	1.8	(0.5, 6.7)	0.365	1.6	(0.4, 6.7)	0.491
4	General symptoms	2.5	(0.4, 16.5)	0.314	0.4	(0.1, 2.5)	0.323	5.0	(0.8, 30.8)	0.081	2.4	(0.8, 7.6)	0.123
4	EET symptoms	0.5	(0.1, 3.0)	0.400	1.1	(0.3, 4.1)	0.920	1.7	(0.5, 5.8)	0.370	1.5	(0.5, 4.3)	0.434
4	GI symptoms	0.7	(0.2, 2.2)	0.517	0.7	(0.2, 2.5)	0.561	0.5	(0.1, 1.8)	0.247	1.1	(0.3, 4.5)	0.887

*Denotes statistical significance, based on an alpha level = 0.05.

© 2023 Doll MK et al. *JAMA Network Open.*

eFigure 1. Flow Diagram Detailing Enrollment and Outcomes in the COVIDIPS Cohort

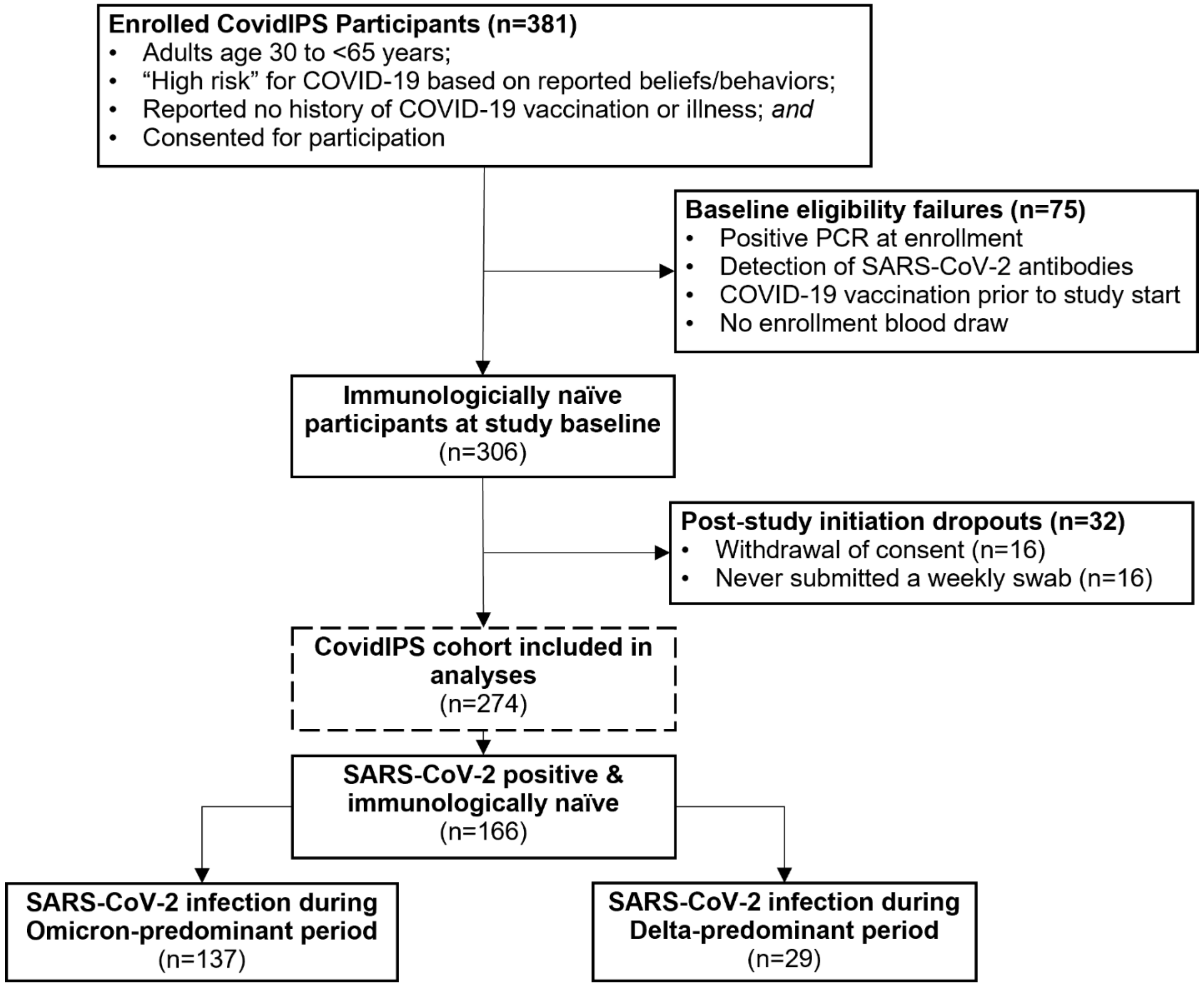

© 2023 Doll MK et al. *JAMA Network Open.*

eFigure 2. Prevalence of Acute COVID-19 Symptoms, Excluding General Symptoms, by Variant

Data represents the percentage of symptoms reported among participants who submitted a survey each day; only symptomatic persons were included (n=155). *Abbreviations*: EET: eyes, ears, and throat; GI: gastrointestinal.

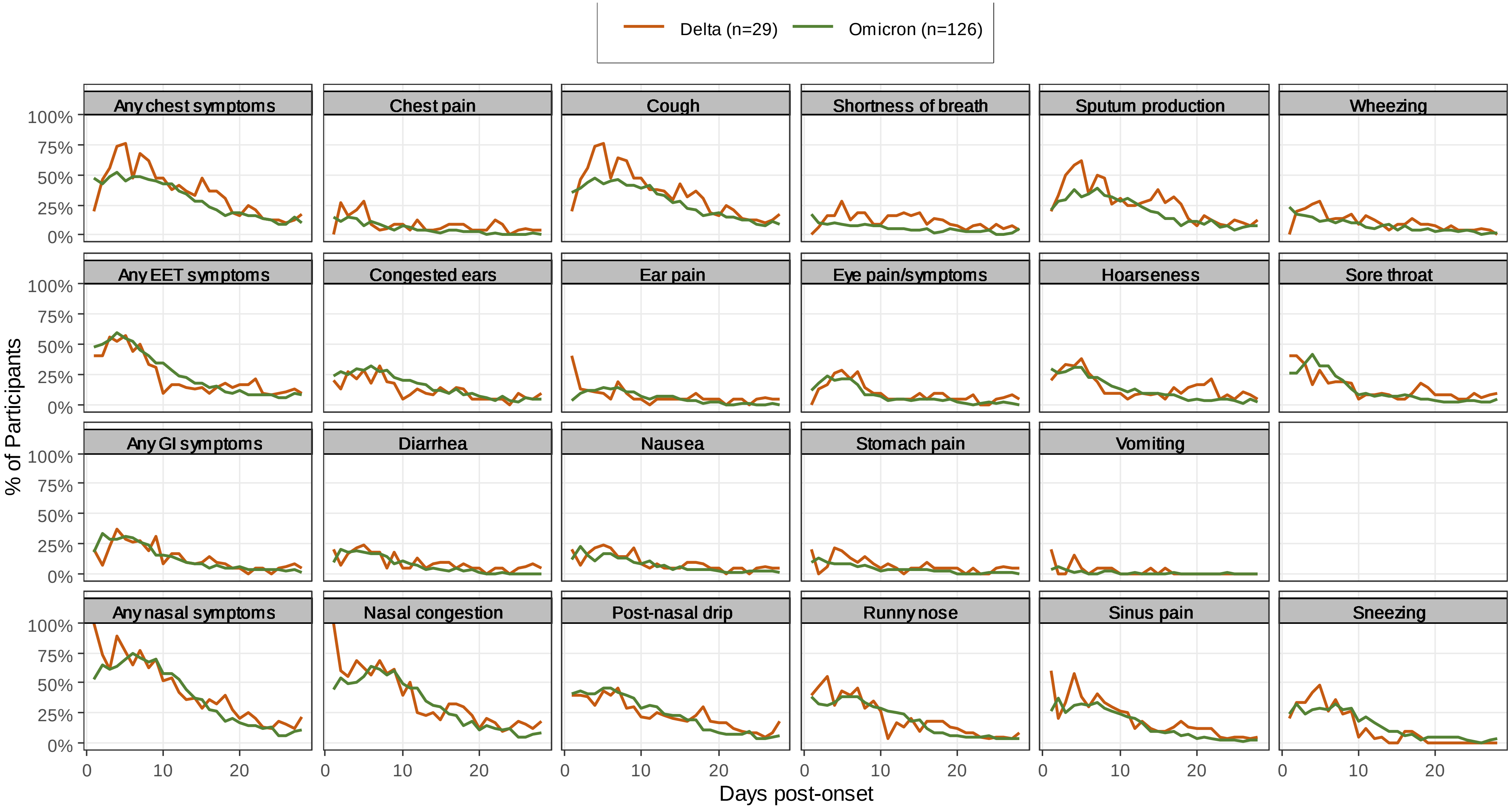

© 2023 Doll MK et al. *JAMA Network Open.*

eFigure 3. Mean Number of Acute Symptoms by Day and Variant

Data represents the mean severity rating for each symptom among participants who submitted a survey each day; only symptomatic persons were included (n=155).

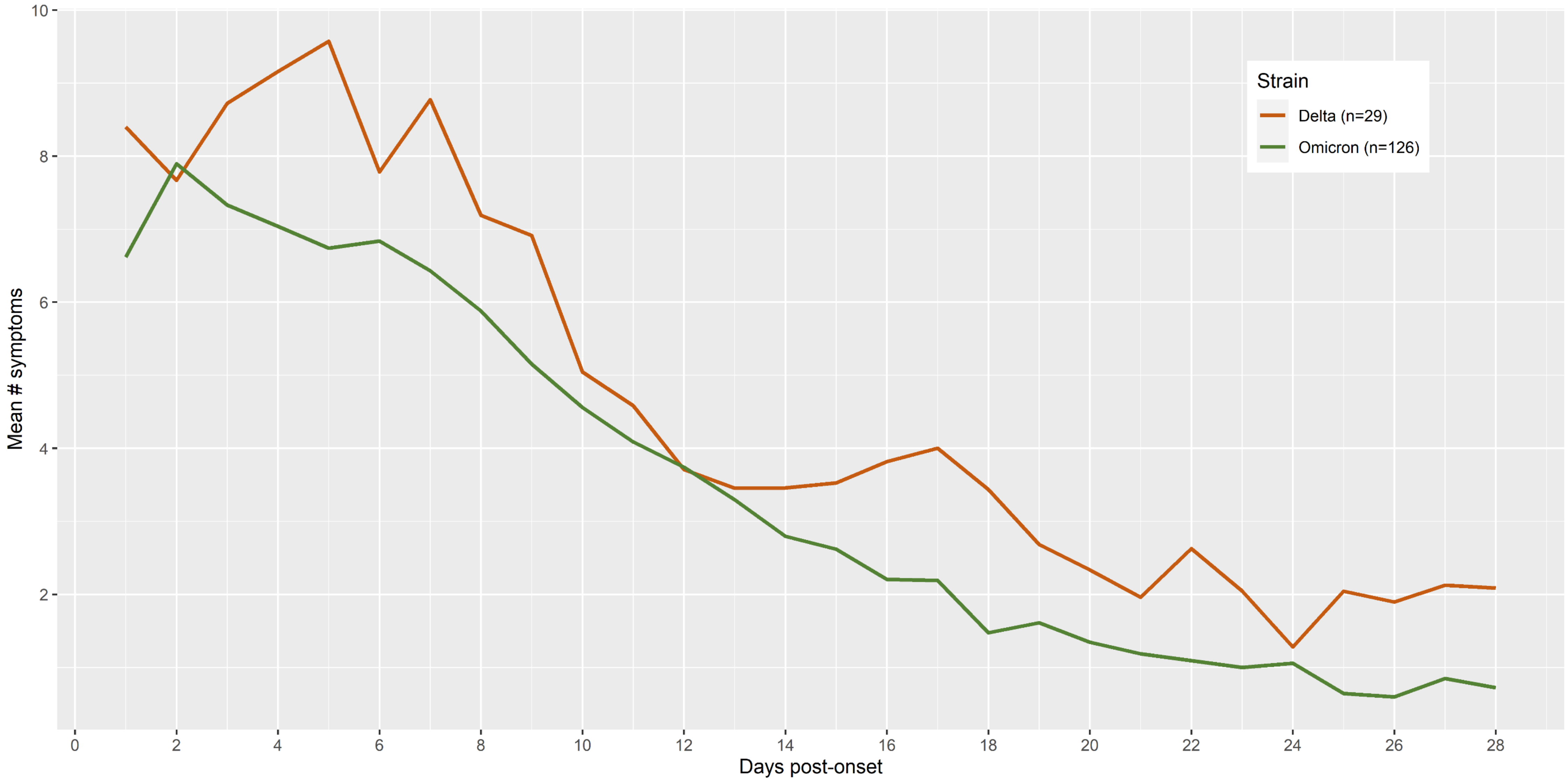

© 2023 Doll MK et al. *JAMA Network Open.*

eFigure 4. Mean Severity of Postacute COVID-19 Symptoms by Variant

Data represents the mean severity rating for each symptom among participants who submitted a survey each week; only persons who ever reported any postacute symptoms were included (n=33). *Abbreviations*: EET: eyes, ears, and throat; GI: gastrointestinal.

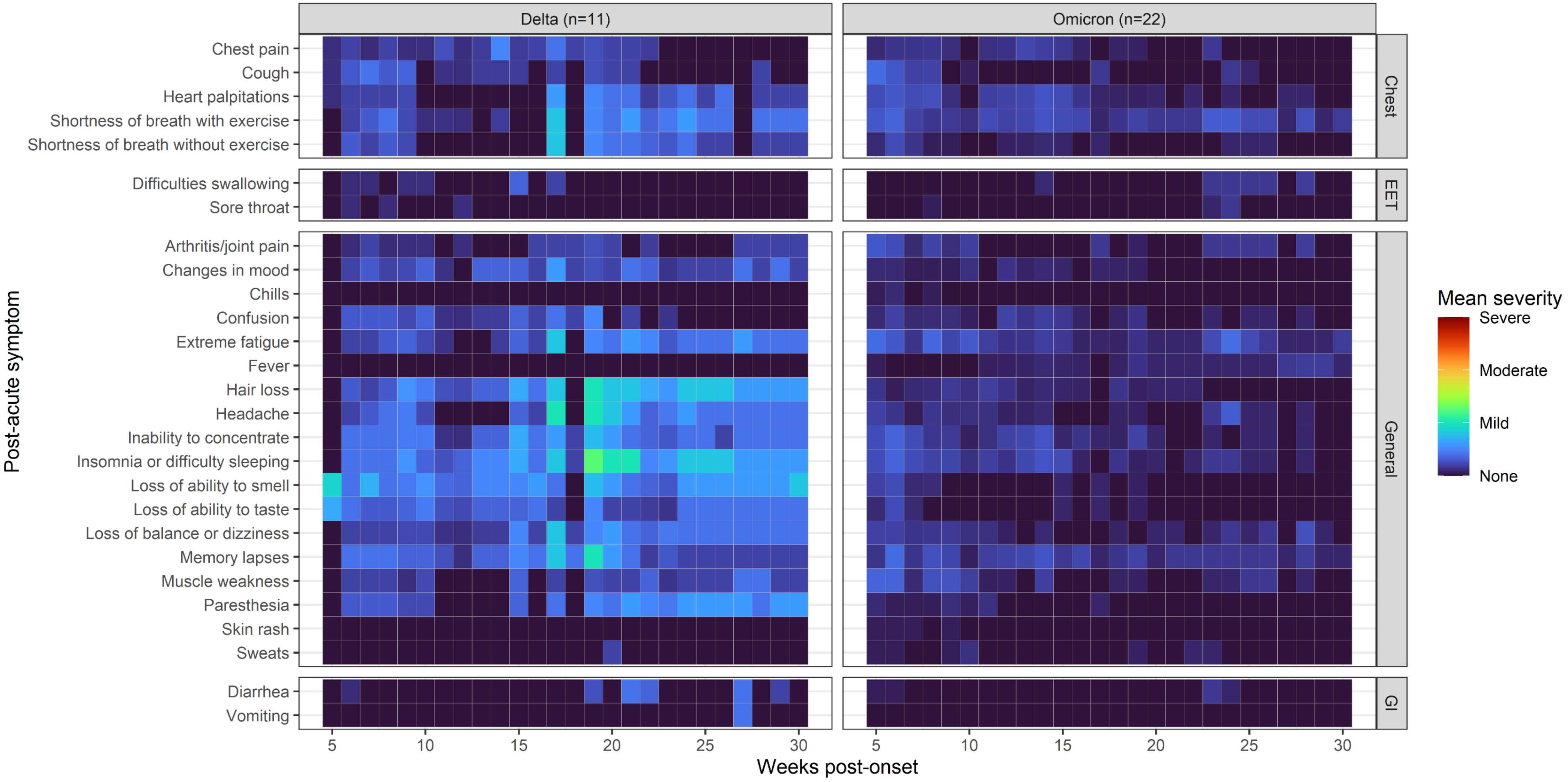

© 2023 Doll MK et al. *JAMA Network Open.*

eFigure 5. Trajectory of Postacute COVID-19 Symptoms by Variant

Each box represents the total number of symptoms reported (max 28) by participants who ever reported postacute symptoms (n=33) during the postacute period (≥5 weeks). Shaded grey values represent weeks the participant did not contribute data because the time period was outside the study window (or between primary/extension periods); while missing data due to survey non-response is represented by black “X”s.

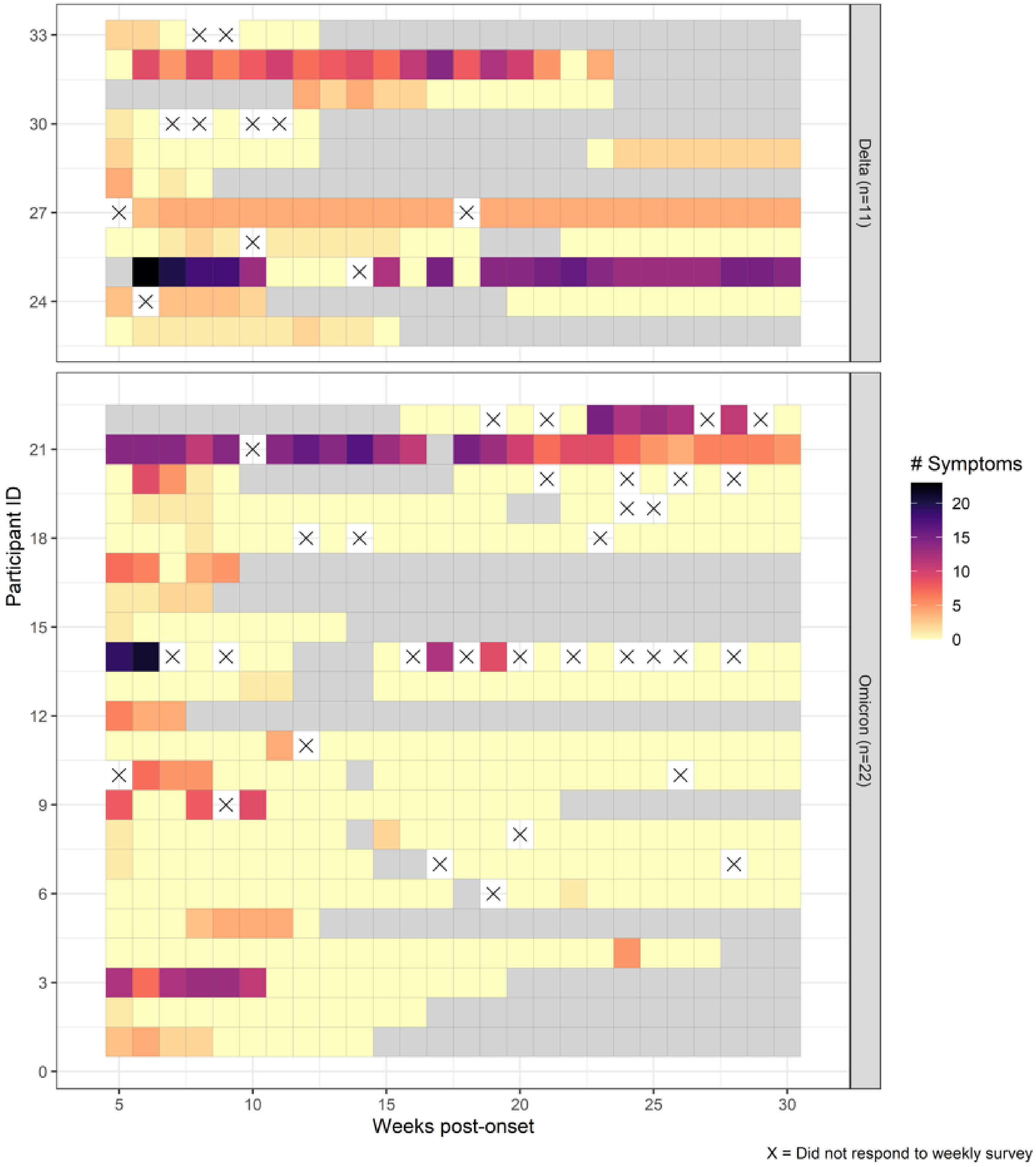

© 2023 Doll MK et al. *JAMA Network Open.*

eFigure 6. Prevalence of Acute COVID-19 Symptoms by Postacute Status

Data represents the percentage of symptoms reported among participants who submitted a survey each day; only unvaccinated and persons with one episode of SARS-CoV-2 were included (n=142). *Abbreviations*: EET: eyes, ears, and throat; GI: gastrointestinal.

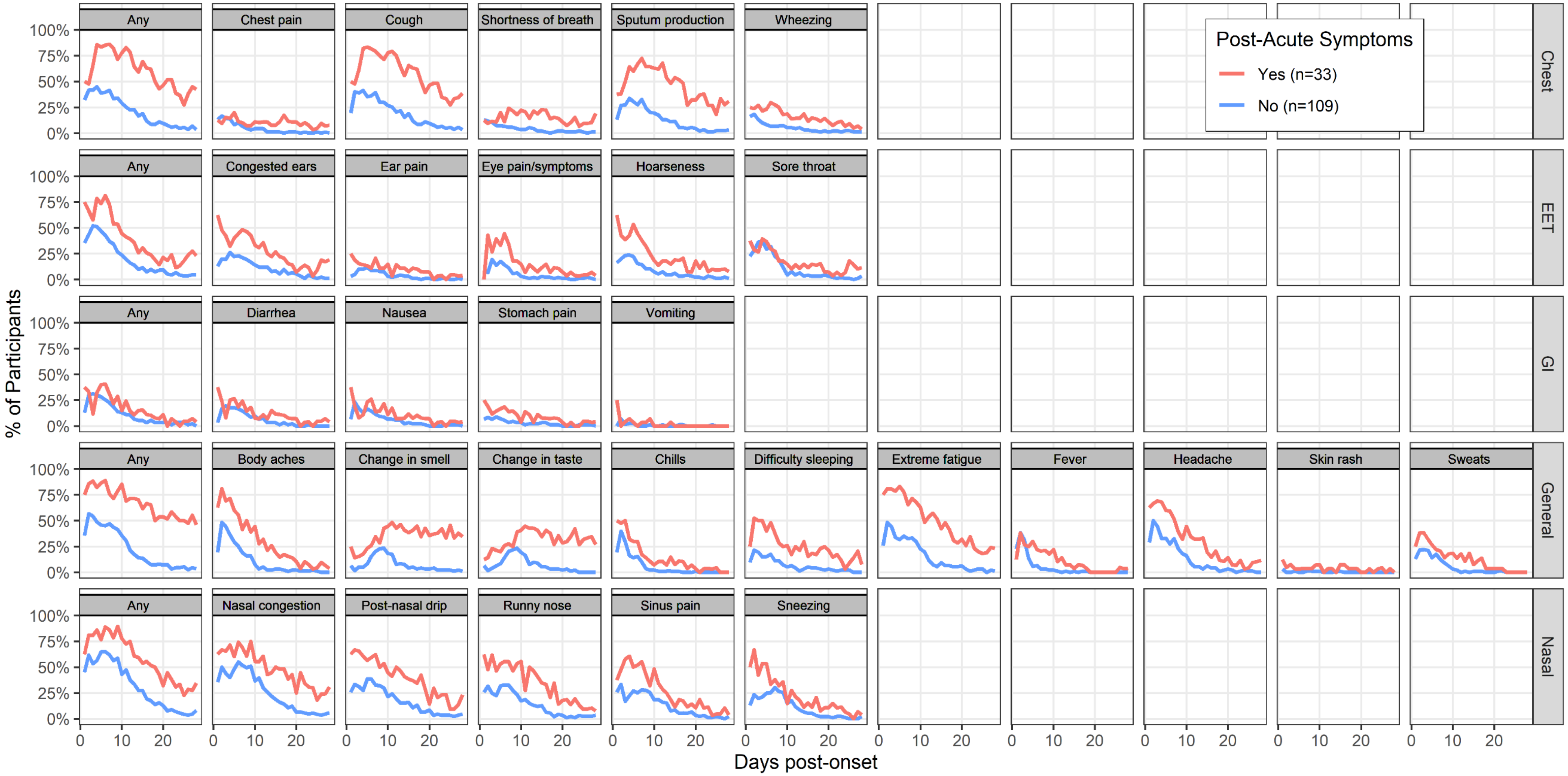

© 2023 Doll MK et al. *JAMA Network Open.*

eFigure 7. Mean Severity of Acute Symptoms by Day and Postacute Status

Data represents the mean severity rating for each symptom among participants who submitted a survey each day; only unvaccinated and persons with one episode of SARS-CoV-2 were included (n=142). *Abbreviations*: EET: eyes, ears, and throat; GI: gastrointestinal.

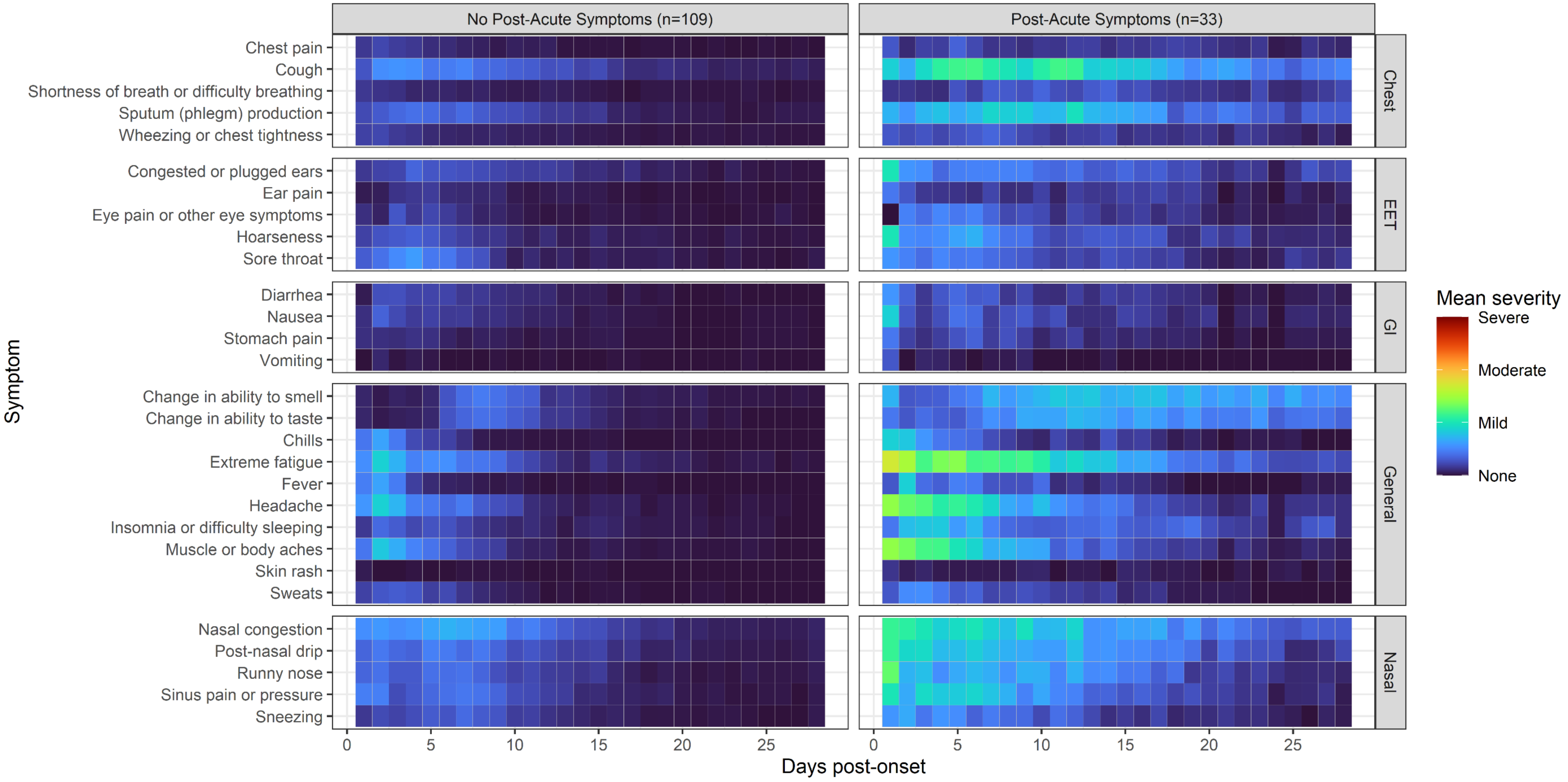

© 2023 Doll MK et al. *JAMA Network Open.*
