# Supplementary material for: Acute and Postacute COVID-19 Outcomes Among Immunologically Naive Adults During Delta vs Omicron Waves
Source: JAMA Netw Open. 2023 Feb 28;6(2):e231181. doi: 10.1001/jamanetworkopen.2023.1181 (PMC9975921; doi:10.1001/jamanetworkopen.2023.1181)
Supplement: Supplement 2. — Data Sharing Statement [file jamanetwopen-e231181-s002.pdf]

Data Sharing Statement

Doll. Acute and Postacute COVID-19 Outcomes Among Immunologically Naive Adults During Delta vs Omicron Waves. *JAMA Netw Open*. Published February 28, 2023.
doi:10.1001/jamanetworkopen.2023.1181

Data
Data available: No
